# What is eDNA method standardisation and why do we need it?

**DOI:** 10.3897/mbmg.9.132076

**Published:** 2025-02-13

**Authors:** Susanna Theroux, Adam Sepulveda, Cathryn L. Abbott, Zachary Gold, Alison W. Watts, Margaret E. Hunter, Katy E. Klymus, Shana Lee Hirsch, Joseph M. Craine, Devin N. Jones, Rachel J. Brown, Joshua A. Steele, Miwa Takahashi, Rachel T. Noble, John A. Darling

**Affiliations:** 1 Southern California Coastal Water Research Project, Costa Mesa, CA, USA; 2 U.S. Geological Survey, Northern Rocky Mountain Science Center, Bozeman, MT, USA; 3 Fisheries and Oceans Canada, Nanaimo, British Columbia, Canada; 4 NOAA Pacific Marine Environmental Laboratory, Seattle, WA, USA; 5 University of New Hampshire, Durham, NH, USA; 6 U.S. Geological Survey, Wetland and Aquatic Research Center, Gainesville, FL, USA; 7 U.S. Geological Survey, Columbia Environmental Research Center, Columbia, OH, USA; 8 University of Washington, Seattle, WA, USA; 9 Jonah Ventures, Boulder, CO, USA; 10 Whitney Genetics Laboratory, US Fish and Wildlife Service, Onalaska, WI, USA; 11 Commonwealth Scientific and Industrial Research Organisation, Crawley, Australia; 12 Institute of Marine Sciences, UNC Chapel Hill, Morehead City, NC, USA; 13 Center for Ecological Measurement & Modeling, US Environmental Protection Agency, Research Triangle Park, NC, USA

**Keywords:** Environmental DNA, lab accreditation, management, methods, standardisation

## Abstract

The rapid advancement of environmental DNA (eDNA) science in the past two decades has inspired a concomitant growth in the development of eDNA sampling and analytical methods. However, these methods are often developed by individual laboratories or institutions, which can isolate protocols within programmes, agencies or regions and prevent the beneficial exchange of data and ideas. Recent efforts to advance national and international coordination have resulted in a groundswell of standardisation efforts, but there is still considerable confusion around the role of formal standards for regulatory or research applications. With this commentary, we hope to provide clarity on the terminology used in standardisation discussions, including the differences between formal standards and best practice guidelines. Additionally, we discuss how eDNA method choice may be informed by environmental management scenarios and review examples of formal eDNA method standards being used to inform management action. The eDNA community now has an opportunity to develop a roadmap for method development to help close standardisation gaps, advance eDNA method adoption and accelerate our ability to monitor biological life at the scales our current environmental challenges demand.

## Introduction

Environmental DNA (eDNA) methods have revolutionised the way we are able to monitor and assess biological communities (Baird and Hajibabaei 2012; Chavez et al. 2021; Pawlowski et al. 2021). The ability to detect organisms at trace levels, differentiate morphologically indistinct taxa and survey multiple phylogenetic groups from a single sample is providing researchers with a depth of biological data that was previously unattainable (Gold et al. 2021; Keller et al. 2022; Preston et al. 2024). A growing body of research in the past two decades has helped to establish eDNA-based methods (hereafter, ‘eDNA methods’) as a critical component of biological research (Bohmann et al. 2014; Taberlet et al. 2018; Beng and Corlett 2020), used to assess the presence of invasive or endangered species, detangle multitrophic food networks and evaluate community responses to restoration or anthropogenic impacts (Sepulveda et al. 2019; Gold et al. 2021; Keller et al. 2022; Allan et al. 2023; Pont et al. 2023; Preston et al. 2024).

There is now a growing desire to transition eDNA methods from research to application or ‘translation to practice’ (Blackman et al. 2024), for informing environmental management decision-making. Standardised eDNA methods are a critical component of this transition, as they provide a solid foundation of scientific consensus and help establish decision-maker and stakeholder trust in the results (Ferrante et al. 2022; Bunholi et al. 2023). Standardised methods help to ensure that data meet accepted levels of quality control and assurance (Trujillo-González et al. 2021) and that resulting datasets can be integrated and compared (Wilson-Wilde 2018). Standardised methods can also have an important catalytic effect on markets for eDNA technologies, providing potential service providers with license to operate and potential customers with reason to trust them (Lodge 2022). The general lack of standardised eDNA methods across agencies and jurisdictions has been cited is a primary impediment to regulatory uptake of eDNA methods (Trujillo-González et al. 2021; Blancher et al. 2022; Kelly et al. 2023).

The importance of method standardisation was evident during the development of protocols for SARS-CoV-2 wastewater surveillance. The urgency required by the global health crisis meant that sampling and testing protocols were developed in the absence of standardised methods (Servetas et al. 2022), resulting in over 35 protocols for quantifying the SARS-CoV-2 genetic signal in raw wastewater (Pecson et al. 2021). This lack of method standardisation limited method availability, consistency and comparability of resulting monitoring data as well as the ability to compare results generated from different protocols and laboratories (Servetas et al. 2022). Thankfully, there has been movement towards greater standardisation of SARS-CoV-2 wastewater surveillance methods (Oyervides-Muñoz et al. 2024) and data reporting (McClary-Gutierrez et al. 2021) in recent years. In addition to wastewater surveillance, there are other molecular standardisation efforts that can be used as models for advancing eDNA method standardisation, including human forensics (ANSI/ASB 2018, 2019; Wilson-Wilde 2018), wildlife forensics (SWFS Technical Working Group 2018; Moore et al. 2021; Webster et al. 2024) and the human microbiome (Methé et al. 2012; Stulberg et al. 2016; Greathouse et al. 2019; Amos et al. 2020; Mirzayi et al. 2021). Below, we highlight “bright spots” (Cvitanovic and Hobday 2018) or key examples where eDNA science has influenced policy and practice, such as in the use of eDNA methods for great crested newt surveillance (Biggs et al. 2014).

To pave the way for more bright spots, there is now a call for greater co-ordination and collaboration to advance eDNA methods from research to implementation (Lodge 2022; Bernos et al. 2023; Bunholi et al. 2023; Kelly et al. 2023). To meet this call, we must first take stock of the successful efforts towards eDNA method standardisation and where we need to remove barriers to implementation. The goals of this commentary are to provide semantic clarification of standard terminology commonly used when discussing eDNA methods, provide examples of eDNA method standardisation and coordination efforts and help identify priorities for advancing standardisation efforts to facilitate eDNA methods adoption. We highlight the different types of standardisation strategies that can co-exist, their individual strengths and limitations and the critical need for coordination across political and geographic boundaries to help accelerate eDNA methods standardisation at a global scale.

### Standards terminology

First, it is valuable to clarify what we mean by “standardisation” (Tate and Panteghini 2007; Brunsson et al. 2012). Standardisation is a catch-all term that refers to the process of developing and implementing specifications, based on the consensus of the views of expert users, interest groups and governments (Sherif 2006; Saltzman et al. 2008). The resulting standardised procedures are intended to promote compatibility, interoperability and quality (Xie et al. 2016). A formal “**standard”** or **“de jure standard”** (literally “by law”) is a document developed by subject matter experts and approved by a formal standards organisation that provides guidance on the design, use or performance of materials, products, processes, services and systems (ISO 2024). Formal standard organisations include the International Organisation for Standardisation (ISO), the European Committee for Standardisation (CEN) and national standard bodies, such as the French Association Francaise de Normalisation (AFNOR), the German Institute for Standardisation (DIN), the British Standards Institution (BSI) and the Canadian Standards Association (CSA group). “A laboratory can be “**accredited**” to a formal standard, meaning the laboratory adheres to nationally or internationally recognised standardsards (e.g. ISO/IEC 17025 2017) and has been assessed as competent by a formal accreditation body, such as the American National Standards Institute National Accreditation Board (ANSI 2024).

**Figure 1.**
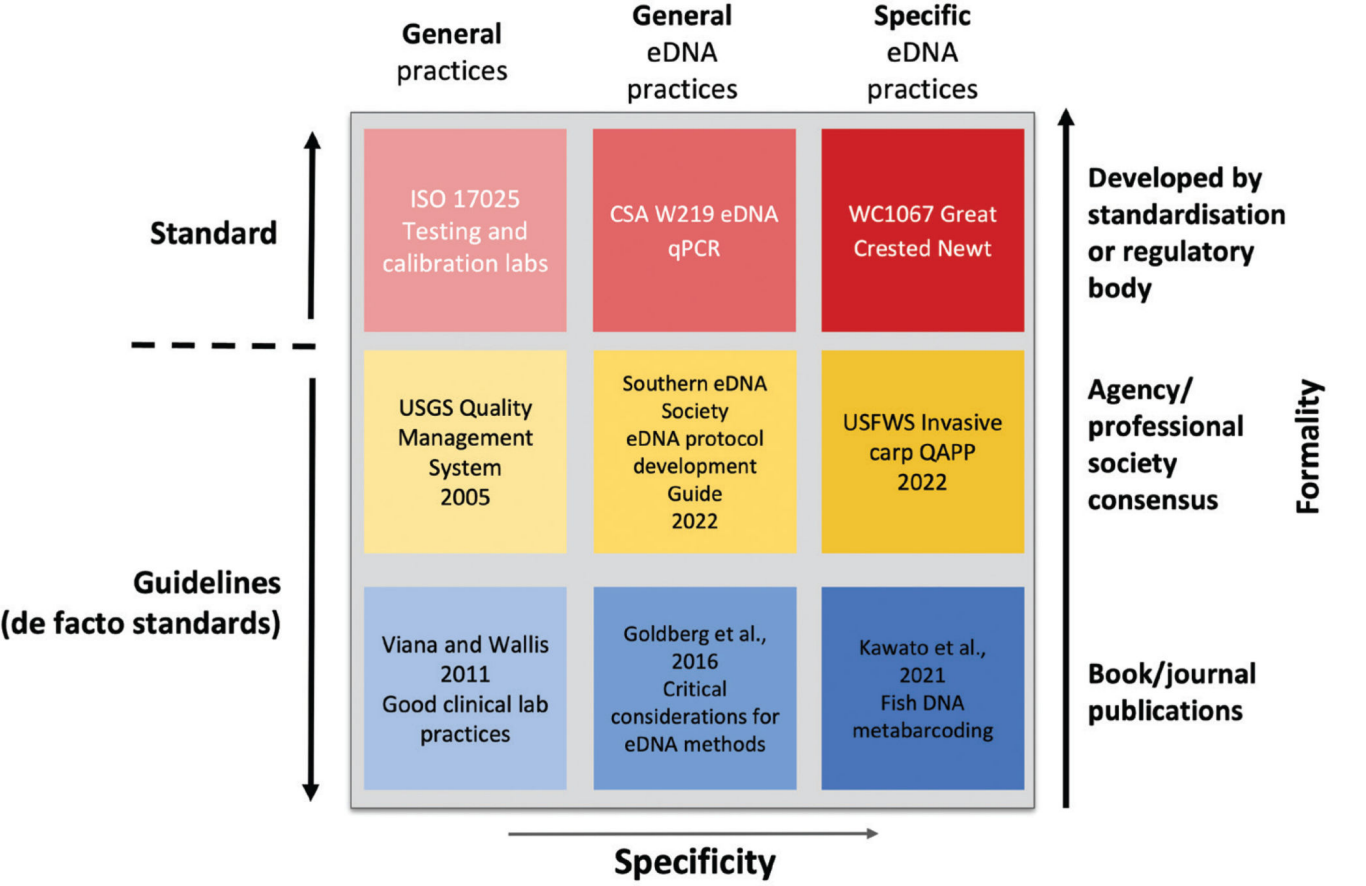
Overview of method standardisation depicting the difference between standards and guidelines, with examples for each tier of formality and specificity. The term ‘standards’ is reserved for those documents that are created by standardisation bodies. The term ‘guidelines’ applies to all other documents related to best practices and procedures.

Alternatively, a “**de facto standard”** (literally “of fact”) is a document that can likewise provide requirements, specifications and characteristics to be used consistently in a method procedure, but is developed outside of a formal standards organisation, by groups such as professional organisations (e.g. Southern eDNA Society), federal agencies (e.g. U.S. Fish & Wildlife Service) or via peer review (Doi et al. 2021). Although they lack formal accreditation, de facto standards are often derived from professional consensus, undergo multiple levels of testing and validation and can serve to provide information for formal standards. For eDNA applications, we opt to use the phrase “**guidelines**” when referring to de facto standards, as it helps further delineate the two types of standards with greater clarity (Fig. 1).

What applications require a formal standard? In general, accreditation to a formal standard is pursued by laboratories, either research or commercial, generating data that will be used in a regulatory context or for informing high-risk management decisions (e.g. fisheries management). Precision, accuracy and repeatability are key priorities in these scenarios, as are defensibility and traceability (Sepulveda et al. 2020; Webster et al. 2024). It should be noted that some countries (e.g. France) mandate laboratory accreditation and use of formal standard methods (Zima 2017). In contrast, many research laboratories may be satisfied using well-validated best practice guidelines to generate high-quality data without the investment in laboratory accreditation to a formal standard. Pursuing lab accreditation is a non-trivial, expensive process (Trujillo-González et al. 2021) and, therefore, it is not necessarily suitable for all laboratories and end-users (Lee et al. 2024).

Formal standards can range from general lab practices to more specific, even species-specific, DNA assays procedures (Fig. 1). For example, the general primary lab standard ISO 17025 establishes that a laboratory has an acceptable quality management system in place and has the ability and competence to provide testing and calibration results (ISO/IEC 17025 2017). For many diagnostic applications, ISO 17025 is required together with accreditation to a more specific topical standard (e.g. ISO 9308 enumeration of Escherichia coli bacteria). More specific to eDNA methods, the national standard CSA W219 establishes performance criteria for targeted qPCR analyses of eDNA samples in Canadian laboratories (Canadian Standards Association 2023). Likewise, Natural England’s national formal standard WC1067 pertains to qPCR-based analyses for the presence or absence of great crested newts *Triturus cristatus* (Biggs et al. 2014), an endangered species in the United Kingdom but not in other parts of Europe. Formal standard ISO EN 17805 ‘Water quality – Sampling, capture and preservation of environmental DNA from water’, preceded by the European CEN standard 17805 (CEN EN 17805 2023), is currently under development and is slated to become the first international eDNA standard (ISO/DIS 17805 2024), hopefully setting the stage for future standards development.

Like standards, guidelines can range from general to specific (Fig. 1). Recommendations on good clinical laboratory practices for molecular tests used in diagnostic laboratories (Viana and Wallis 2011) cover general lab practices and establish systems of quality management and quality assurance. Likewise, the U.S. Geological Survey has detailed quality management systems guidelines and processes that laboratories must adhere to (USGS 2005). More specific to eDNA methods, the Southern eDNA Society protocol development guide (De Brauwer et al. 2022) and the seminal paper on critical considerations for eDNA methods (Goldberg et al. 2016) provide best practice guidelines geared towards eDNA applications. Lastly, the USFWS invasive carp (*Hypophthalmichthys* spp.) quality assurance project plan (USFWS 2022) and the fish DNA metabarcoding guidelines from Kawato et al. (2021) provide specific guidelines on eDNA methods and procedures.

### Standardisation and coordination efforts

In the past decade, multiple national eDNA strategies and roadmaps have been developed to outline key goals towards eDNA method development and standardisation. An example of roadmaps and strategies include those developed in Finland (Norros et al. 2022), France/Switzerland (Lefrançois et al. 2018), Australia/New Zealand (De Brauwer et al. 2023), Japan (The eDNA Society 2019) and the United States (National Aquatic Environmental DNA Strategy 2024). Below, we highlight additional organisations that are focused on method coordination and standardisation that play a critical role in helping to break down silos amongst jurisdictions, agencies and researchers (Table 1).

At the regional scale, the California Molecular Methods Workgroup (MMWG 2024) uses consensus-based approaches to develop guidelines and standardised operating procedures that bring greater consistency and comparability to DNA-based analyses for regional biomonitoring programmes. The Workgroup is comprised of representatives from water quality management agencies, researchers, tribal communities, and wastewater and stormwater discharge agencies and also functions as a training body to increase eDNA literacy amongst interested user communities. At the national scale, the U.S. Government Environmental DNA Working Group (GeDWG) is a ‘no-cost consortium’ that brings together federal, state, provincial, municipal and other government and non-government agencies interested in eDNA methods development and application, with monthly meetings and an annual workshop that focuses on sharing technical expertise and increasing eDNA method awareness (Stepien et al. 2023).

In the European Union, the DNAqua-Net initiative (COST Action CA15219) helped expedite the development of standardised eDNA protocols for biomonitoring and bioassessment, including sampling and analyses of diatoms (CEN/TR 17244 and 17245 (2018); Kelly (2019), Vasselon et al. (2021); Baricevic et al. (2022)) and water eDNA samples (CEN EN 17805 (2023); Hering et al. (2018); Bruce et al. (2021); Blancher et al. (2022)) and serves as an exemplar of the power of coordinated, collaborative standardised methods development. At the international scale, the recently formed International eDNA Standardisation Task Force (iESTF) is a multinational group focused on developing and proposing standard seed documents to ISO (iESTF 2024). Ultimately, these international standards are needed to bridge differences amongst regional standards and to allow for broad applicability and inclusivity. Notably, recent national (Stepien et al. 2023) and international (Yamanaka et al. 2023) workshops and conferences focused on transitioning eDNA methods to management implementation have helped to increase collaboration amongst these organisations to reduce redundancy and streamline standardisation.

### Priorities for advancing methods standardisation

There are a few key areas where eDNA method standardisation efforts would benefit from additional focus. As eDNA methods continue to be developed and adopted, laboratory proficiency testing and intercalibration exercises will be required to help ensure that methods are being implemented correctly (Blackman et al. 2019; Trujillo-González et al. 2021). However, a key component of these exercises is the availability of standardised reference material (SRM) to evaluate method performance. Currently, eDNA researchers are limited to microbial reference material (e.g. ZymoBIOMICS Microbial Community DNA Standard) and there are no publicly-registered producers of certified reference materials (ISO/IEC 17034 2016) or proficiency testing providers (ISO/IEC 17043 2023) that can assess the competence and reliability of eDNA-based workflows for macrobial targets (Trujillo-González et al. 2021). Future investments in standardised reference materials for multicellular organisms will provide critical mechanisms to evaluate eDNA method performance and further advance eDNA method adoption.

Another key consideration in the adoption of eDNA applications for management decisions is the rigidity or flexibility allowed by eDNA standards, i.e. should standards define process or should they define outcomes? Standards can be written in a way that is highly prescriptive of inputs, dictating each step, kit and reagent. Alternatively, performance-based standards specify outcome criteria that allow for multiple methodologies to produce results that meet established quality measures (e.g. DNA yield for DNA extraction; Kelly 2019). On one hand, rigidity in eDNA standards helps to minimise errors and biases and helps to facilitate the integration of datasets (Wilson-Wilde 2018). On the other hand, flexibility in eDNA standards allows for broader engagement and adaptation to changing methods and technology.

As with any method standardisation, there is a risk that eDNA method standards result in “lock-in” (Lee et al. 2024), wherein a selected method or protocol prescribes a technology or process that is outdated within a few years. It is valuable to remember that “while standards and standardisation are typically associated with stability and sameness, they are essentially a dynamic phenomenon” (Brunsson et al. 2012). In this vein, standards developed by ISO are revisited every few years to update as necessary (Boiral 2011). A recent review of nine years of data generated using the great crested newt WC1067 standard method (Biggs et al. 2014) recommended potential modifications in DNA extraction method to accommodate technological advancements (Rees et al. 2023). Environmental DNA sampling and analytical techniques are undoubtedly going to advance in the next decade, especially as agency adoption helps to facilitate commercial investment and technological innovation (Lodge 2022). It will be up to programme and agency leads to establish processes to update and revise standards on a routine basis and to determine how such processes will accommodate technological innovation and avoid lock-in.

Finally, there is a critical need for the eDNA field to spotlight ethical considerations of eDNA sampling and data sharing that will provide opportunities for diverse user communities to participate in eDNA research, while protecting data sovereignty and privacy (Jennings et al. 2023). Environmental DNA standardisation should promote, not interfere, with the inclusion of community scientists, indigenous communities and communities with limited resources. The Global South has been under-represented in many international standardisation efforts and the field of eDNA would benefit from broader participation and inclusion (Curtis et al. 2023; Hirsch et al. 2024). The recent call for a voluntary, global moratorium on the genetic analysis of individual humans, based on human genetic material derived from eDNA samples (Doi and Kelly 2023), further underscores the importance of establishing ethical guidelines that are sensitive to human rights. A coordinated, international effort focused on these ethical considerations would help further the adoption of these methods in a safe, consistent manner and will ensure the continued growth, maturity and adoption of eDNA methods for decades to come.

## Figures and Tables

**Table 1.** A subset of regional, national and international eDNA methods standardisation efforts.

Geographic scope	Name	Region	Method standardisation	Lab accreditation/Proficiency testing	Coordination	Other	Link/ref
US-based	Invasive Carp Regional Coordinating Committee	US	x	x	x	Database & reporting	https://invasivecarp.us/
	READI-Net	US	x	x	x	Database & reporting	https://www.usgs.gov/search?keywords=READI-Net
	Marine Environmental DNA (eDNA) Technology Committee	US	x		x	Workshops	https://mtsociety.memberclicks.net/marine-environmental-dna-technology-committee
	USGS Nonindigenous Aquatic Species (NAS)	US	x			Database & reporting	https://nas.er.usgs.gov/
	Government eDNA Working Group (GEDWG)	US			x		Stepien et al. (2023)
	California Molecular Methods Workgroup (MMWG)	CA	x		x		https://mywaterquality.ca.gov/monitoring_council/mmw.html
	CALeDNA	CA	x			Bioinformatics	https://ucedna.com/
	Estuary eDNA	NERR					https://www.estuarydna.org/
	West Coast Ocean Biomolecular Observing Network (OBON)	US West Coast	x		x	R&D	https://evsatt.github.io/WC-OBON_Website/
	NOAA ‘Omics Working Group	US	X		X	R&D, Database & reporting, Bioinformatics	https://oceanexplorer.noaa.gov/technology/omics/noaa-omics.html
	US Fish and Wildlife Service	US	x				https://www.fws.gov/eDNABMP_FWS_2023
Regional	DNAquaNet	Europe	x		x		http://dnaquahub.eu/
	eDNAquaPlan	Europe	x		x	Database, reference libraries	https://ednaquaplan.com/
	DNAquaMG	Europe	x		x		https://dnaquaimg.eu/
	iTrackDNA	Canada	x	x	x		https://itrackdna.ca/
	Southern eDNA Society	Australia/NZ	x		x		https://sednasociety.com/
	eDNA Society	Japan					https://ednasociety.org/en/
	AfricaBioGenome	Africa	x		x	Genome sequencing	https://africanbiogenome.org/
Global	International eDNA Standardisation Task Force (iESTF)	Global	x		x		https://iestf.global
	Better Biomolecular Ocean Practices (BeBOP)	Global	x		x	Metadata reporting	https://oceandecade.org/actions/better-biomolecular-ocean-practices/
	Marine Biodiversity Observing Network (MBON)	Global			x		https://marinebon.org/
	Ocean Biomolecular Observing Network (OBON)	Global					https://www.obon-ocean.org/
	eDNA Collaborative	Global			x	R&D	https://www.ednacollab.org/
	Global Biodiversity Information Facility (GBIF)	Global	x			Metadata reporting	https://docs.gbif.org/publishing-dna-derived-data/en/
	International Barcode of Life (iBOL)	Global			x	Reference barcodes	https://ibol.org/

## Data Availability

All of the data that support the findings of this study are available in the main text.
